# Supplementary figures and images for: Erythropoietin restrains the inhibitory potential of interneurons in the mouse hippocampus
Source: Mol Psychiatry. 2024 Apr 15;29(10):2979–96. doi: 10.1038/s41380-024-02528-2 (PMC11449791; doi:10.1038/s41380-024-02528-2)

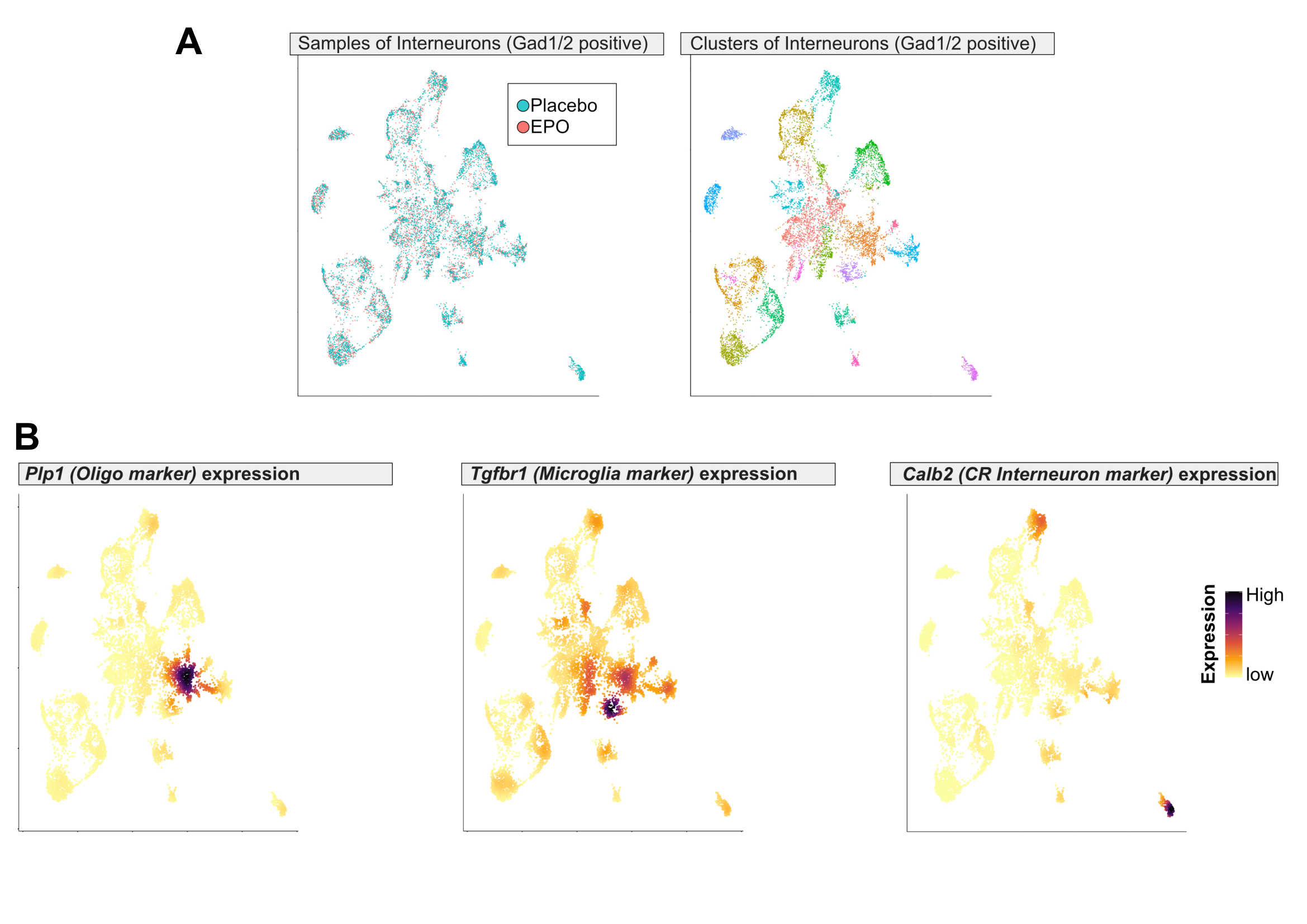

Supplement: Supplementary file 1 — Supplementary Figure 1 [file 41380_2024_2528_MOESM1_ESM.tif]

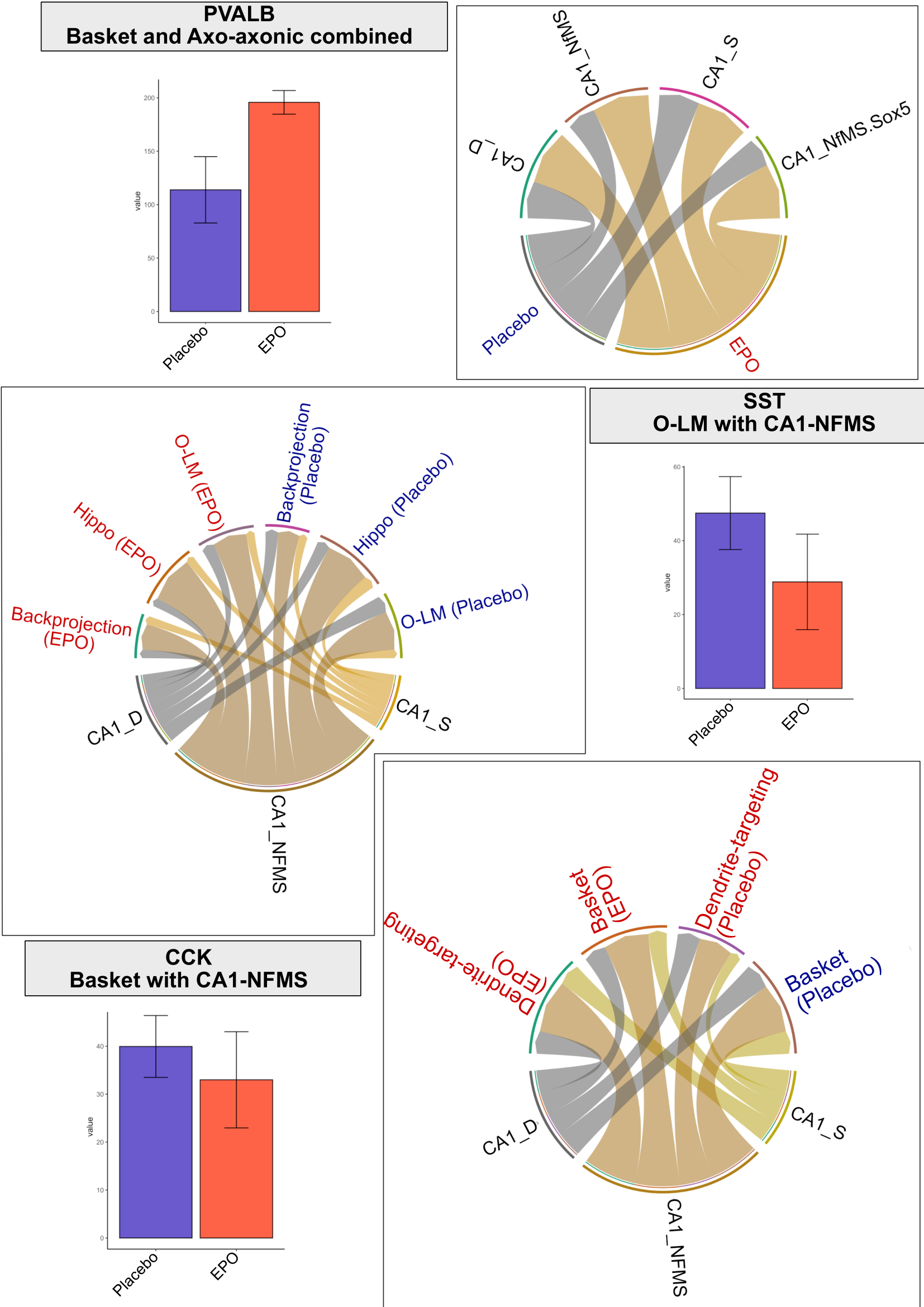

Supplement: Supplementary file 2 — Supplementary Figure 2 [file 41380_2024_2528_MOESM2_ESM.tif]
